# Molecular Model of the Microvillar Cytoskeleton and Organization of the Brush Border

**DOI:** 10.1371/journal.pone.0009406

**Published:** 2010-02-24

**Authors:** Jeffrey W. Brown, C. James McKnight

**Affiliations:** Department of Physiology and Biophysics, Boston University School of Medicine, Boston, Massachusetts, United States of America; George Mason University, United States of America

## Abstract

**Background:**

Brush border microvilli are ∼1-µm long finger-like projections emanating from the apical surfaces of certain, specialized absorptive epithelial cells. A highly symmetric hexagonal array of thousands of these uniformly sized structures form the brush border, which in addition to aiding in nutrient absorption also defends the large surface area against pathogens. Here, we present a molecular model of the protein cytoskeleton responsible for this dramatic cellular morphology.

**Methodology/Principal Findings:**

The model is constructed from published crystallographic and microscopic structures reported by several groups over the last 30+ years. Our efforts resulted in a single, unique, self-consistent arrangement of actin, fimbrin, villin, brush border myosin (Myo1A), calmodulin, and brush border spectrin. The central actin core bundle that supports the microvillus is nearly saturated with fimbrin and villin cross-linkers and has a density similar to that found in protein crystals. The proposed model accounts for all major proteinaceous components, reproduces the experimentally determined stoichiometry, and is consistent with the size and morphology of the biological brush border membrane.

**Conclusions/Significance:**

The model presented here will serve as a structural framework to explain many of the dynamic cellular processes occurring over several time scales, such as protein diffusion, association, and turnover, lipid raft sorting, membrane deformation, cytoskeletal-membrane interactions, and even effacement of the brush border by invading pathogens. In addition, this model provides a structural basis for evaluating the equilibrium processes that result in the uniform size and structure of the highly dynamic microvilli.

## Introduction

In order to facilitate exchange between the extracellular milieu and the intracellular cytosol, the absorptive epithelium of the gastrointestinal tract and the renal proximal convoluted tubule have developed a highly specialized apical membrane, termed the brush border, which provides a ∼30-fold increase in surface area over a similarly sized planar surface. This brush border is composed of a hexagonal array of uniformly sized, finger-like projections, called microvilli.

Polyacrylamide gel electrophoresis of isolated brush borders demonstrated that this large macromolecular complex is primarily composed of only six protein components, which were later identified as actin, fimbrin, villin, brush border myosin (Myo1A), calmodulin, and a non-erythrocytic spectrin (Reviewed by Mooseker [1]). Briefly, ∼19 actin filaments, cross-linked by fimbrin and villin, serve as the “core bundle,” which is laterally tethered to the adjacent membrane through myosin1A:calmodulin cross-bridges. This apparatus has been reconstituted *in vitro* [2]. As each core bundle enters the apical cytoplasm, it is secured and hexagonally arranged by the terminal web, which is composed of a non-erythrocytic spectrin.

Although individual microvilli are amotile, persistent, uniformly sized structures, their underlying cytoskeleton is highly dynamic. The entire macromolecular complex is turned over every ∼20 minutes [3]. Also, in response to cellular signaling, stress, and specifically increases in intramicrovillar Ca^2+^, villin converts from an F-actin bundling protein to an F-actin severing protein, causing the dissolution of the underlying cytoskeleton and collapse of the microvillus [4]. As a plus-end directed myosin, Myo1A is continuously undergoing powerstrokes [5], which preferentially sort lipid rafts and their associated proteins to the tip of the microvillus, where they are more accessible to luminal contents. Further, these powerstrokes create membrane tension, which likely acts synergistically with a “Brownian Ratchet” mechanism [6] of plus-end actin monomer addition at microvillar tips to deform the membrane into the dramatic morphology of the brush border.

In addition to being essential for nutrient uptake, the apical brush border is a key portal for intestinal pathogens and every cytoskeletal protein component of the host's microvillus plays an essential role in the pathogenesis of one organism or another. *Salmonella spp.* (*S. typhimurium*, typhoid fever; *S. enterica*, gastrointestinal enteritis) secrete SipA into the host cell, where it binds actin [7], [8], [9] and increases the bundling efficiency of fimbrin [10]. As part of its pathognomonic attaching and effacing mechanism, enteropathogenic *Escherichia coli* (infantile diarrhea) secrete EspB, which binds Myo1A and prevents its association with actin [11]. This interaction presumably eliminates the lateral cross-bridges between the membrane and the core bundle, destroying the microvillus [12]. The pathogenicity of *Shigella flexneri* (bacillary dysentery) is dependent on villin, as villin knockout mice are completely resistant to infection by this organism [13]. In addition to bacteria, protozoa like *Entamoeba histolytica* (amebic colitis and amebic liver abscesses) also take advantage of host microvillar proteins [14].

Since 1950, when Granger and Baker reported the first electron micrographs of the gastrointestinal epithelium [15], the structure of the brush border, the microvillus, and their individual protein components have been the focus of a great number of studies. Here, we combine the reconstructions of each of the individual components bound to actin into a single, unique, self-consistent model of the microvillar and brush border cytoskeleton.

## Results

### The Paracrystalline Hexagonal Array of F-actin Filaments

**Figure 1:**
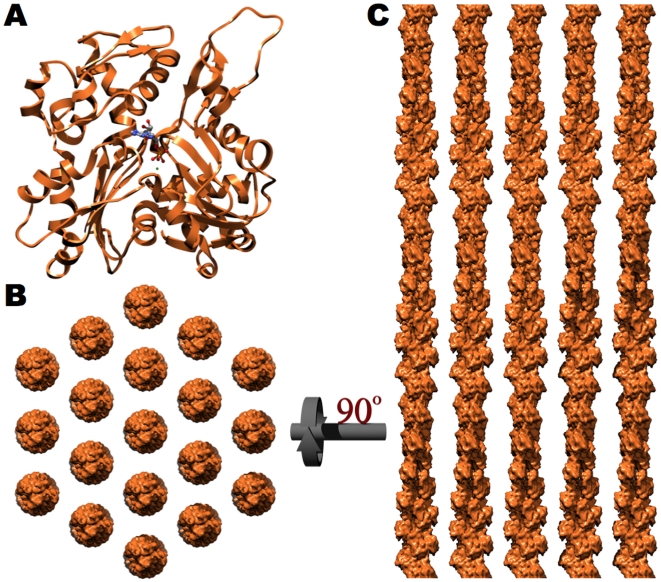
Paracrystalline arrangement of actin microfilaments within the microvillar core bundle. **A**. Ribbon diagram of an actin monomer with its associated nucleotide (PDB ID: 2ZWH) [16]. The microvillar core bundle is formed through the parallel, lateral association of 19 actin filaments. **B**. When viewed down the long axis of the bundle, the filaments are hexagonally arranged with a center-to-center spacing of 12.0 nm. **C**. A side view of the actin core bundle, rotated 90° with respect to **B**, displays the unipolar orientation (pointed or minus end up) and axial alignment of each microfilament.

The equilibrium between actin's monomeric (globular, G-actin) and multimeric (filamentous, F-actin) form is dependent on the concentration of actin, salt, and a great number of actin binding proteins. The actin microfilament is formed through the association of actin monomers (Fig. 1A) into a double helix with ∼13/6 symmetry [16] (Nomenclature explained in Fig. S1). Approximately 19 of these actin filaments laterally assemble into a hexagonal array with a center-to-center spacing of 12.0 nm (Fig. 1B, C). Diffraction studies have demonstrated that actin's 13/6 symmetry is retained within native microvillar core bundles [17], which, as will be discussed below, has important consequences for the organization of its associated F-actin binding proteins.

This core bundle possesses paracrystalline order as each filament is unipolar, with its barbed (plus) end embedded in the dense plaque atop each microvillus [18], and in axial register [19], [20], meaning that the cross-over points of all filaments are precisely aligned with one another (Fig. 1C). This paracrystalline order is not solely observed of the microvillar cytoskeleton but is also present in the aural stereocilia [21] and is believed to be a general characteristic of bundled microfilaments [22].

The placement of actin's 13/6 helical geometry into a hexagonal lattice imposes specific demands on a cross-linker because the 13/6 helical geometry differs slightly from an ideal hexagonal geometry (i.e. a 6/1 helix). This mismatched symmetry requires that the protein cross-linkers are slightly flexible such that they able to accommodate a ±7° deviation from their ideal binding orientation. However, the presence of a single, unique cross-linking site per actin repeat (defined here as 13 actin monomers) dictates that the cross-linker's flexibility must be less than ±14°. If the flexibility of either fimbrin or villin was greater than ±14°, one would have expected to observe both the genuine binding conformation as well as its reciprocal (180° rotation, in which the two actin binding domains swap their respective filaments) in the two dimensional arrays [23], [24].

### The Primary F-Actin Cross-Linking Protein, Fimbrin

**Figure 2 pone-0009406-g002:**
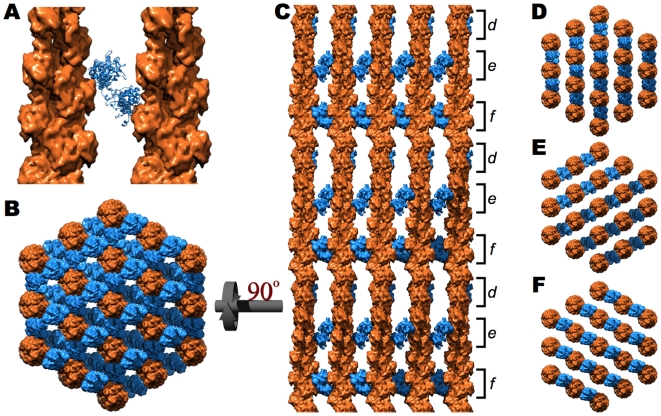
The fimbrin cross-linked core bundle [23]. **A**. Ribbon diagram of fimbrin (blue) cross-linking two actin filaments (orange surfaces). **B**. When viewed down the long axis of the bundle, fimbrin cross-links exist between every adjacent pair of microfilaments. **C**. A side view, rotated 90° with respect to **B**, displays the three distinct vertical levels (d, e, and f) of fimbrin cross-links corresponding to the three different directions of fimbrin cross-links (**D**, **E**, and **F**, respectively). The slight irregularity in the vertical orientation of d, e, and f is a consequence of cross-linking actin's 13/6 symmetry within a hexagonal lattice.

The structure of fimbrin, the protein responsible for the paracrystalline order of the microvillus core bundle, in a two-dimensional array with F-actin has been reported (Fig. 2A) [23]. Importantly, these 2D crystals possess the same geometric parameters present in the three-dimensional microvillar core bundle, namely, the actin filaments are unipolar, in axial register, and have center-to-center spacing of 12.0 nm and, therefore, are almost certainly representative of the fimbrin cross-links within the native microvillar core bundle.

In a similar approach to that described by Volkmann and colleagues [23], extension of this two dimensional array into the three dimensional core bundle was accomplished through parallel, axially aligned sheets of actin and fimbrin, where every other sheet is staggered by one half of the interfilament spacing in order to achieve hexagonal symmetry (Fig. 2D-F). The precise axial alignment of the actin filaments ensures that, with a vertical offset, all three arrangements of parallel fimbrin:F-actin arrays are equivalent with respect to their actin filaments and, therefore, all fimbrin cross-links may coexist (Fig. 2).

The vertical offset of fimbrin cross-links is a consequence of F-actin's helicity. In a helical polymer, vertical translation rotates the direction at which each actin (and fimbrin binding site) points. In order to cross-link adjacent filaments, fimbrin requires that its two binding sites on adjacent filaments are across from one another. The fact that all actin filaments are unipolar and in axial register is important because this allows each protein's binding site on different filaments to rotate in phase with one another. Therefore, when viewed from the side, the vertical position of the fimbrin cross-links is dependent upon the relative orientation of the two microfilaments being cross-linked (Fig. 2C-F).

### The Secondary F-Actin Cross-Linking Protein, Villin

In addition to fimbrin, a second cross-linking protein, villin, exists within the microvillar core bundle. However, unlike fimbrin cross-linked actin bundles which are indistinguishable from intact microvillar core bundles, those formed with villin are looser and less-well organized [17], [25]. The hypothesis that villin's cross-linking activity is subordinate to that of fimbrin, is supported by the presence of microscopically normal microvilli despite its absence in the villin knockout mouse [26].

A three dimensional reconstruction of villin cross-linking two actin microfilaments was determined by analyzing 2D arrays of actin and villin [24]. Despite significant heterogeneity in the interfilament spacing, vertical offset, and roll of the filament, Hampton *et al*. were able to identify how the two distinct F-actin binding domains in villin [(1) V_1–6_, composed of 6 gelsolin-like repeats and (2) the C-terminal headpiece domain] associate with their respective filament and how villin's individual domains are organized with respect to one another [24]. In order to position villin into the microvillar core bundle; however, the model must be slightly altered because the model that Hampton and others settled on had an interfilament spacing that is slightly wider than that measured of intact microvillar core bundles (12.6 nm versus 12.0 nm) and contains a 1.7 nm offset.

**Figure 3 pone-0009406-g003:**
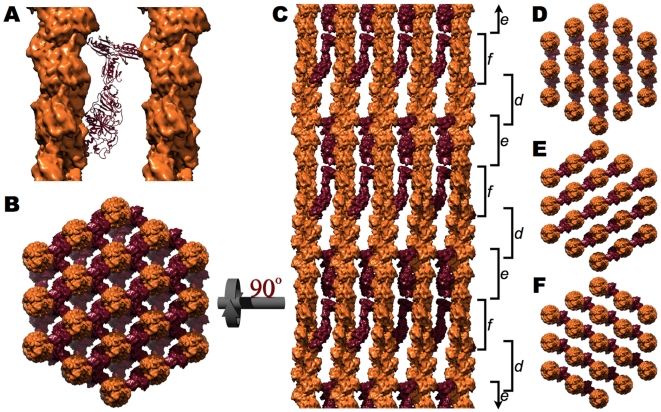
The villin cross-linked core bundle [24]. **A**. Ribbon diagram of villin (maroon) cross-linking two actin filaments (orange surfaces). **B**. When viewed down the long axis of the bundle, a villin cross-link exists between every adjacent pair of microfilaments. **C**. A side view, rotated 90° with respect to **B**, displays the three distinct vertical levels (d, e, and f) of villin cross-links corresponding to the three different directions of the villin cross-links (**D**, **E**, and **F**, respectively). The slight irregularity in the vertical orientation of d, e, and f is a consequence of cross-linking actin's 13/6 symmetry within a hexagonal lattice.

These differences were remedied by separately docking each of villin's two F-actin binding domains onto the actin core bundle and, as suggested by Hampton and colleagues, remodeling the unstructured linker which connects these two domains (Fig. S2). The resulting vi`llin cross-linked core bundle is displayed in Figure 3. The unstructured linker may explain why, compared to fimbrin, microfilament bundles cross-linked by villin are less well organized [17], [25]. Importantly, the position of villin cross-links do not compete with those of fimbrin for F-actin (Movie S1).

### Myosin1A Laterally Tethers the Core Bundle to the Adjacent Membrane

**Figure 4:**
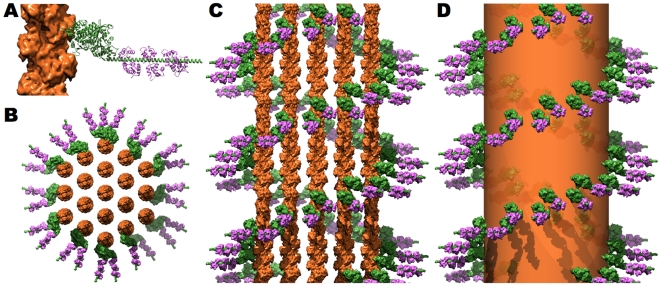
Structure of the myosin 1A, calmodulin cross-bridges. **A**. Ribbon diagram of brush border myosin (green) [78] in a near rigor conformation with its three associated calmodulin light chains (purple) [38] bound to actin (orange surface). **B**. When viewed down the long axis, two to three Myo1A:CaM cross-bridges radially extend out from each outer filament in the core bundle. **C, D**. When viewed from the side one may appreciate the barber-pole like motif of Myo1A:Calmodulin cross-bridges about the actin core bundle (depicted as orange molecular surfaces in **C** and as a transparent orange cylinder in **D**).

The microvillar core bundle is laterally tethered to the adjacent microvillar membrane by a brush border specific, non-filamentous isoform of myosin, Myo1A (Reviewed in [27] and [28]). Both electron microscopic reconstructions of Myo1A decorating microfilaments and biophysical assays have demonstrated that this protein is a fully functional plus-end directed myosin [5], [29] and that it binds actin in a manner indistinguishable from that of conventional, class-II myosins [30], [31]. The only significant morphological differences between Myo1A and conventional myosins are that (1) Myo1A lacks the first ∼70 residues which fold into a Src-Homology 3 domain, (2) Myo1A has a significantly longer powerstroke, and (3) that its rigor conformation is nearly perpendicular to the actin microfilament (Fig. 4A) [30], [31].

When Myo1A is radially positioned about the core bundle, it forms a double helical barber pole-like structure (Fig. 4B-D) because the precise axial alignment of each microfilament radially transfers the double helical symmetry of the central actin filament to the outer ring of microfilaments. When Myo1A is modeled onto the outer ring of actin filaments, it becomes apparent that two myosins are able to associate with each repeat along an outer microfilament, which is consistent with and explains the experimentally determined stoichiometry [32]. A third myosin can be positioned on a single actin filament per 360° turn about the outside of the bundle (Fig. 4B). All other positions are either sterically prohibited (due to clashes with adjacent microfilaments and cross-linkers) and/or do not exhibit adequate radial extension to simultaneously bind the core bundle and the microvillar membrane. Interestingly, in a hexagonal arrangement of 19 actin filaments, movement between adjacent outer filaments is synonymous with translating one actin monomer up or down the long helix of actin (Fig. 4B-D). The barber pole-like arrangement of Myo1A has been directly visualized and provides very strong evidence for the precise axial alignment of actin filaments within the core bundle because no other organized arrangement of microfilaments can result in this motif [19], [20].

Myo1A, as well as all class-1 myosins, has a highly basic Tail Homology (TH1) domain located at its C-terminus that binds negatively charged phospholipids [33]. The TH1 domain of Myo1A preferentially associates with lipid rafts present in the microvillar membrane [34], [35] that contain at least one class of negatively charged phospholipid, phosphatidylserine [36]. Despite the lack of a high-resolution structure for this domain, its approximate shape and dimensions can be obtained from either three-dimensional helical reconstructions of negatively stained Myo1A decorating F-actin [31] or two-dimensional crystals of Myo1A on negatively charged phospholipids [37].

### Myosin 1A's Regulatory Calmodulin Light Chains

Three regulatory calmodulin light chains associate with an equal number of tandem IQ domains along the alpha helical neck of Myo1A [28]. Houdusse *et al*. recently reported the structure of two calmodulin light chains bound to a tandem pair of IQ domains along the neck domain of Myosin V [38]. Importantly, the sequences of these IQ domains are very similar to those present in Myo1A and the spacing between them is identical.

As illustrated in Figure 4, the calmodulin light chains nearly completely envelop the length of the alpha helical neck domain and likely stabilizes the single alpha helix against the bowing strain that it experiences during a powerstroke. Although this level of modeling is able to provide a sense of how these calmodulin light chains stabilize the alpha helical neck region, it is not likely to be of sufficient accuracy to explain how the three, calmodulin light chains regulate Myo1A's kinetics.

### The Microvillar Cytoskeleton *In Situ*

**Figure 5:**
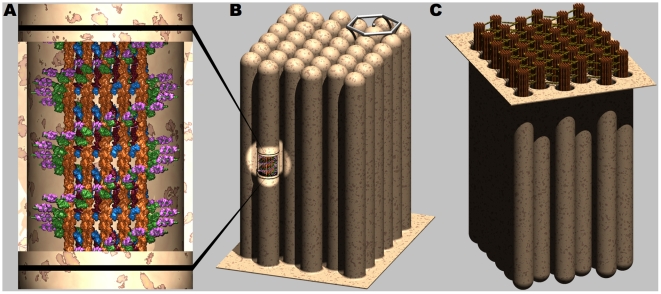
The microvillar cytoskeleton *in situ*. **A**. When our modeled cytoskeleton is enveloped with a membrane of appropriate dimensions, it is apparent that the proposed model of the microvillar cytoskeleton precisely spans the ∼100 nm diameter required to establish a circumferential connection to the membrane. The color irregularity of membrane is an “Artistic License” employed to emphasize the importance of lipid raft domains within the brush border membrane (Reviewed in [84]). **B**. The relative size of the microvillar cytoskeleton with respect to the brush border may be appreciated when the microvilli are hexagonally arranged as they exist within the brush border [26]. **C**. A schematic representation of the terminal web, in which multiple spectrin tetramers (α-spectrin, brown; β-spectrin, yellow) cross-link and hexagonally arrange the microvillar core bundles (depicted here as orange molecular surfaces) as they enter the apical cytoplasm. Within the microvillus, the barbed end of actin is positioned towards the apex, and therefore the vertical orientation of actin in **A** and **B** is reversed relative to how actin is traditionally viewed (pointed end up; Figures 1–4).

Micrographs of transversely sectioned brush borders illustrate the strict hexagonal packing of individual microvilli across the apical surface of the enterocyte. In mice, Fourier analysis of these micrographs established the center-to-center spacing to be 115–120 nm [26]. After subtracting the radii of two core bundles (∼50–55 nm depending on orientation), we are left with a distance of 60–70 nm between core bundles for the spectrin cross-links in the terminal web of the apical cytoplasm (Fig. 5C).

Although the spectrin tetramer is typically cited as being 200–240 nm in length based primarily on rotary shadowed micrographs [39], [40], a careful review of the literature reveals several lines of evidence which suggest that its biologically functional length is much shorter (∼65–80 nm) and that the extended form is likely a consequence of the *in vitro* conditions employed to isolate and study this protein (low temperature, low ionic strength, and removal of the associated membrane). The simplest evidence for this shorter length is predicated on the numerical density of spectrin tetramers (or equally valid junctional complex components) per surface area of the erythrocyte membrane. Using a consensus value of 10^5^ spectrin tetramers per erythrocyte [41], [42] and assuming a homogeneous hexagonal distribution of junctional complexes across the erythrocyte membrane (surface area of 135 µm^2^), one obtains a value of ∼70 nm length for each spectrin tetramer. Similarly short lengths have been reported from both electron [43], [44], [45] and atomic force [46], [47] microscopic examination of erythrocyte membranes. Furthermore, Hirokawa and colleagues were able to show that within the terminal web, non-erythrocytic spectrin is also considerably shorter than its rotary shadowed length of ∼265 nm [48], [49], [50].

From the previously reported dimensions of individual microvilli and the brush border, we were able to construct an *in silico* model of the apical membrane in order to visualize our modeled protein cytoskeleton as it would exist *in situ* (Fig. 5A,B). As evidenced in Figure 5A, the radial extension of myosin is well-suited for establishing a circumferential connection between the actin core bundle and the microvillar membrane. The relative size of the modeled cytoskeleton to the brush border may be appreciated when microvilli are hexagonally arranged as they exist across the apical surface of the enterocyte (Fig. 5B). The precise hexagonal arrangement of the individual microvilli [26] has been attributed to the terminal web composed of non-erythrocytic spectrin, which cross-link adjacent core bundles as they enter the apical cytoplasm (Fig. 5C).

## Discussion

### Remarkable Symmetry of the Microvillus and Brush Border

The majority of both soluble and membrane bound proteins form homo- and heteromeric macromolecular complexes, which confer genetic, allosteric, and several physicochemical advantages over a similarly large structure formed from a single peptide chain (reviewed in [51]). However, the brush border is an extreme example of a symmetrical apparatus in both the paracrystalline order exhibited by the actin core bundle and the immense size of the complex, which encompasses the entire apical membrane of the enterocyte and therefore the vast majority of the small intestine.

### The F-Actin, Fimbrin, and Villin Core Bundle

Densitometric quantitation of SDS-PAGE separated proteins from demembranated microvilli resulted in the molar ratios of 1.3∶10 and 1.6∶10 for fimbrin:actin and villin:actin, respectively [32]. Assuming complete saturation of all actin cross-linking sites in our hexagonal array of 19 filaments, one obtains a ratio of 1.7∶10 of these two proteins to actin. The similarity between the experimental value and that predicted by our model corroborates the hypothesis that both fimbrin and villin crosslink actin filaments through a single, non-mutually exclusive position and that *in vivo* their binding sites are nearly fully occupied. The ability of fimbrin and villin to simultaneously crosslink two microfilaments is imparted by their disparate binding sites on actin, which, when considering the helical nature of the actin polymer, vertically staggers the two cross-linking sites (Movie S1).

Recently, Galkin and colleagues have presented an alternative model for the fimbrin cross-link based on aligning a crystal structure of fimbrin to their three dimensional reconstruction of fimbrin's second actin binding domain (ABD2) decorating actin [52]. When their proposed model is positioned within the actin core bundle, we find that, although the 3D arrangement of the individual CH domains is different from that used in our model [23], the vertical position of these cross-links is very similar (compare Fig. 2 to Fig. S3). Close inspection reveals that the reason for the similar cross-linking position is that the footprint of the third CH domain on actin (the only CH domain in their alternative model that significantly contacts actin) is essentially the same in the two structures, albeit a different interface is contacting actin. The study by Galkin and colleagues suggests that, *in isolation*, the two CH domains, which comprise ABD2, may bind actin in manner distinct from that of the full-length construct. We prefer and have used the fimbrin model from Volkmann *et al*. because it is the actual three-dimensional reconstruction of full-length fimbrin cross-linking two microfilaments.

Figure S2 compares the structure of villin cross-linking two actin filaments separated by 12.6 nm and offset by 1.7 nm [24] to our proposed model of villin as it would exist within the microvillus core bundle (12.0 nm with no offset). As realized and suggested by Hampton *et al*., 2008, elimination of the offset would result in dissociation of a presumably weak interaction between V_2_ and its adjacent filament [24]. Further, translating the two, actin filaments with respect to one another changes the relative position of the two F-actin binding domains. This is accounted for in our model by simply remodeling the long, unstructured linker domain between V_1–6_ and the headpiece domain [53]. The close proximity of villin headpiece and V_4–6_ in our model is consistent with a study demonstrating a calcium sensitive interaction between these two domains [54]. Further, when the interfilament spacing is reduced to that observed in the microvillus core bundle (12.0 nm), V_6_ comes in close proximity to the actin filament. This suggests that the second actin-binding surface in villin likely includes contributions from both the headpiece domain as well as from V_6_. This hypothesis is supported by the recent report of a “cryptic” actin-binding site located within V_6_ [53].

Based on data demonstrating that, unlike F-actin bundles cross-linked with fimbrin which are very similar to those of microvillar core bundles, actin filaments cross-linked with villin are looser and less well-organized [17], [25], we conclude that villin's cross-linking activity is subordinate to that of fimbrin. This hypothesis is supported by the presence of microscopically normal microvilli despite the absence of villin in the knockout mouse [26]. Although it has been suggested that as a redundant cross-linking protein, villin might result in stronger, better organized core bundles [55], more recent experiments argue that the definitive function of villin is the dynamic reorganization of the core cytoskeleton in response to cell signaling and stress [26].

Unlike fimbrin, which is a relatively inert F-actin bundling protein, villin is unique in that it switches from an F-actin bundling protein to an F-actin severing and capping protein when subjected to low µM concentrations of Ca^2+^ [4], [56], [57], [58]. This functionality appears to be important for the dissolution of the brush border after either prolonged fasting or increases in intracellular calcium [26]. Furthermore, ingestion of chemicals noxious to the gastrointestinal epithelium resulted in greater mortality in villin-null animals [26].

Although it has been reported that the Ca^2+^-dependent severing activity of villin is augmented by phosphatidylinositol 4,5-bisphosphate (PIP_2_) [59], [60], our model suggests that *as a structural component of the microvillus*, villin is unable to simultaneously interact with both the membrane and the actin core bundle, except possibly at the microvillar tip. Therefore, the activation of villin's *severing activity* by PIP_2_ cannot significantly contribute to dissolution of microvilli. However, as severing and capping are typically coupled activities for actin modifying enzymes including villin [58], the interaction between villin and PIP_2_ at the microvillar tips could potentially cap these filaments, preventing the addition of new actin monomers, which, when considering the continuous treadmilling of actin, would, over the course of ∼20 minutes, extinguish that microvillus.

### The Myosin 1A, Calmodulin Cross-Bridges

Although the use of a myosin to laterally tether the core bundle to its adjacent membrane may at first seem an odd choice, its use confers several advantages over a static cross-bridge. As myosin cycles through its powerstroke, it detaches from and subsequently reattaches to the microfilament. As our model demonstrated that two myosins are able to bind each repeat along an actin filament, the connection between each actin filament and the membrane exhibits significant redundancy, and, therefore, detachment of a single myosin from actin during its powerstroke does not eliminate the connection. The transience of the myosin:actin interaction may be important for the localization of Myo1A within the microvillus because it allows the radially distributed Myo1A proteins to maintain a connection between the core bundle and the adjacent membrane without being affected by incessant downward motion of the treadmilling core bundle.

The Myo1A powerstoke is essential for the cellular localization of lipid raft associated proteins, including sucrase-isomaltase and galectin-4 [61]. As a plus-end directed motor, Myo1A translates lipid rafts and their associated proteins towards the tip of the microvillus where they are more accessible to the luminal contents of the gastrointestinal tract. Furthermore, each powerstroke displaces the membrane towards the tip of the microvillus. Recently, this functionality was dramatically demonstrated by McConnell and Tyska, whose experiments show the ejection of the brush border membrane subsequent to the addition of ATP [5]. These powerstrokes may be directly responsible for the force required to deform the membrane or, more likely, act synergistically with a “Brownian Ratchet” mechanism [6] of actin addition at the tip of the microvillus.

### The Terminal Web

As illustrated by the barber-pole like arrangement of Myo1A about the F-actin core bundle, the precise axial alignment of microfilaments within the core bundle radially transfers the helical geometry of a single actin filament to the outer ring of microfilaments. Therefore the hexagonal arrangement of spectrin and actin in the terminal web of the enterocyte should be viewed as an elaboration of hexagonal spectrin-actin cytoskeleton of the erythrocyte. Whereas the erythrocytic cytoskeleton contains a single actin protofilament and a single spectrin tetramer between each junction complex, the microvillus core bundle is composed of ∼19 actin filaments connected through a web of spectrin cross-links.

### Cytoskeletal Dynamics of the Microvillus

Using GFP-tagged actin, it has been demonstrated that the entire F-actin core bundle is completely turned over every ∼20 minutes, calculated using a value of 0.3 actin/s [3] and a 1,000 nm long microvillus. Further, their data demonstrates that new actin monomers are exclusively added to the barbed end of each microfilament, which is located at the microvillar tip. Similar actin dynamics have been reported in other paracrystalline actin bundles [62]. In order to continuously rebuild the microvillar cytoskeleton, a considerable flux of actin, fimbrin, and villin must occur along the entire length of the microvillus. Further, the incessant treadmilling of actin microfilaments requires that the terminal web is also highly dynamic.

Our model of the saturated core bundle (actin, fimbrin and villin) has a Matthews coefficient of 3.9 Å^3^/Dalton, a value which is within the range of 11,000 protein crystals deposited in the protein data bank (median of 2.52 Å^3^/Dalton) [63]. Unlike small molecules (i.e. absorbed nutrients), which are able to quickly permeate protein crystals, the high density of the core bundle would severely hinder the tipward diffusion of actin, fimbrin and villin and, therefore, these proteins likely travel in the microfilament free zone between the core bundle and membrane to reach the microvillar tip where they are incorporated.

Despite a highly dynamic cytoskeleton, the dimensions of individual microvilli are both uniform and persistent. In comparing microvillar dynamics to that described of other paracrystalline actin bundles [62], the uniform length may be attributed to a dynamic balance between the addition of actin monomers and cross-linkers at the microvillar tip, retrograde translation of the entire complex towards the base, and dissociation of the actin core bundle in the apical cytoplasm. The consistent ∼19 microfilaments present in the core bundle of each microvillus can not be explained by increasing angular disorder of the actin filaments because the primary crossing protein, fimbrin, is also present in stereocilia, which are composed of hundreds to thousands of hexagonally arranged microfilaments. Furthermore, the barber pole motif of Myo1A about the outer actin filaments maintains that these filaments are axially aligned. Therefore, the uniform number of actin microfilaments within each core bundle is likely regulated by the dense plaque located at the apex of each microvillus [64] and which is likely composed of EPS-8 [65] and Myosin 7a [66], among other proteins.

### Unresolved Issues: Small Espin and Ezrin

A third F-actin bundling protein, small espin (∼30 kDa), has been identified in the brush border cytoskeleton [67]. This protein is a splice variant of espin, an F-actin bundling protein found in stereocilia and the protein responsible for the deaf jerker mouse phenotype [68]. However, unlike villin and fimbrin, which are present at levels sufficient to nearly saturate the available cross-linking sites, the molar ratio of small espin to actin is ∼20-fold lower [67]. This suggests that small espin is either localized to one region of the microvillus or is sporadically positioned throughout the actin core bundle. Its low abundance coupled with expression primarily in mature enterocytes, where the brush border has already been established, has led to the suggestion that small espin might simply stabilize preexisting microvilli [67] or may regulate the rate at which actin treadmills [69].

Ezrin, another protein localized to the brush border, was initially believed to laterally tether the core bundle to the membrane; however, this hypothesis was questioned in a recent review [70]. In addition, our model is inconsistent with this conjecture because ezrin, whose structure has been solved [71], is far too small to span the ∼20 nm required to establish this connection. Instead, as demonstrated by its knockout, ezrin is believed to be important in maintaining a connection between the terminal web and the apical membrane [72]. Ultrastructural examination of enterocytes from the ezrin^−/−^ mouse, depict a cytoskeletal protein apparatus similar to that present in wild-type mice; however, it appears to have fallen away from the membrane and as a result only small, non-uniform projections are present on the apical surface of these cells [72].

### Application of This Model

The model presented here will serve as a structural framework to explain many of the dynamic cellular processes occurring over several time scales, such as protein diffusion, association, and turnover, lipid raft sorting, membrane deformation, cytoskeletal-membrane interactions, and even effacement of the brush border by invading pathogens. In addition, this model provides a structural basis for evaluating the equilibrium processes that result in the uniform size and structure of the highly dynamic microvilli.

## Materials and Methods

### Structural Manipulation of Atomic Coordinates

Translations and rotations of peptide chains were carried out with the rotate_pdb program present within the MINRMS suite [73]. All sequence-based backbone alignments were preformed using the molecular graphics program, Friend [74] and structure-based alignments were achieved with the C_alpha_-based algorithm, Topofit [75], also present in Friend. All ribbon and molecular surfaces were calculated with Chimera [76] and exported to POV-RAY [77] where membranes were added and each scene was rendered. All required files as well as instructions to create your own microvillus are available for download at http://people.bu.edu/cjmck/.

### Modeling F-Actin and the Core Bundle

Individual actin microfilaments were constructed in accord with the currently accepted “Holmes model” of the actin filament (PDB ID: 2ZWH) [16]. The 13/6 helical geometry of each microfilament was achieved by translating (27.57 Å) and rotating (−166.154°) each consecutive actin monomer along and about the z-axis. The precise axial alignment of individual microfilaments permits the creation of the core bundle by simply translating individual microfilaments in the x-y plane to those positions corresponding to a hexagonal lattice with a center-to-center spacing of 12.0 nm between adjacent filaments.

### Modeling the Fimbrin Cross-Links

The coordinates of both fimbrin as well as the two, actin filaments that it cross-links were generously provided to us by Niels Volkmann and Dorit Hanein [23].

### Modeling the Villin Cross-Links

Villin cross-links were created from the coordinates of villin [24], generously provided to us by Kenneth Taylor. Two actin filaments were created, which had a center-to-center spacing of 12.6 nm and an offset of 1.7 nm, in accord with the final model reported by Hampton *et al*., 2008. Villin was then carefully positioned between these filaments in visual accordance to that reported by Hampton *et al*., 2008.

### Modeling the Myosin 1A, Calmodulin Cross-Bridges

The actin-binding motor domain of Myo1A was modeled with the crystal structure of myosin 1E (PDB ID: 1LKX) [78], another class 1 myosin with 45% identity to human myosin 1A. The neck domain, which is composed of a single alpha helix, was created by extending the short (6–9 residue) lever arm present in the crystal structure with an ideal alpha helix (created with Moleman2) [79]. As it has been demonstrated that Myo1A binds to F-actin in a manner indistinguishable from that of conventional class II myosins [30], [31], Myo1A was docked to the actin filament through structural alignment of 1LKX to the motor domain of myosin II bound to an actin filament (generously provided by Ken Holmes) [80]. In order to simulate the “strong” binding conformation, the upper domain of 1LKX (residues 132–159, 185–372, 526–548) was excised and structurally aligned to the upper domain in the strong binding conformation of myosin II [80]. The lever arm was rotated and translated to the rigor conformation, which, for Myo1A, is nearly perpendicular to the actin filament [31]. In all figures, the rigor conformation was chosen because it is the conformation of Myo1A under the conditions most commonly employed to study the structure of Myo1A about the actin core bundle.

Calmodulin light chains were added to the Myo1A neck region by aligning the first IQ domain of Myosin V cocrystalized with calmodulin (2IX7, Chains A & B) [38] to each of the three, tandem IQ domains along the alpha helical neck region of Myo1A.

### Arranging Fimbrin, Villin, and Myosin 1A: Calmodulin within the Core Bundle

For every actin binding domain above [Fimbrin: ABD1 & ABD2; Villin: V_1–6_ (residues 17–719) & Headpiece (residues 733–825); Myo1A:3xCaM], there is an associated actin filament. Therefore, each actin binding domain was individually positioned within the microvillus by first structurally aligning its associated actin monomer to the single actin monomer (PDB ID: 2ZWH) used to construct the actin cytoskeleton and then applying all of the translations and rotations, which were previously used to generate every actin monomer in core bundle. From all of the conceivable binding orientations calculated, a single unique position for the fimbrin and villin cross-links were selected using the following criteria: (1) the two, actin-binding domains present in both fimbrin and villin are bound to adjacent filaments and must be located between the two filaments that they are cross-linking, (2) fimbrin's ABD1 should be located towards the pointed end of the actin filament relative to ABD2 and Villin's V_1–6_ should be located towards the pointed end of the actin filament relative to its headpiece domain. Two to three Myo1A:3xCaM complexes per outer filament were selected based on maximizing radial extension and the absence of steric clashes with adjacent microfilament and cross-linkers.

### Modeling Spectrin

An *approximate* model of the spectrin heterotetramer was created with the atomic coordinates of a non-erythrocytic alpha spectrin repeat (PDB IS: 1U4Q) [81] polymerized into a symmetrical, antiparallel double helix with length of 65 nm and pitch and radius in accord with those reported by McGough & Josephs [82]. Spectrin's F-actin binding domain, which is composed of a tandem pair of CH domains located at the N-termini of each beta chain, was modeled using the crystal structure of the homologous domain from alpha-actinin (PDB ID: 2EYI) [83]. It should be noted that arrangement of CH domains in 2EYI are very similar (0.18 nm RMSD) to that of fimbrin (see above).

### Modeling the Microvillar Membrane

The microvillar membrane was created in POV-RAY using the dimensions obtained from electron microscopy of murine brush borders (length, 1000 nm; radius, 50 nm; thickness, 5 nm; hexagonal spacing, 120 nm) [26], [49] and scaled with the molecular surfaces produced by chimera (1 Å per POV-RAY unit). All figures, including those containing multiple peptide chains and/or membranes were rendered orthographically *en bloc*, and, therefore, are true to the 3D atomic model from which they were created.

## Supporting Information

Figure S1 An explanation of F-actin's “13/6” symmetry. A. Surface representation of the F-actin double helix. In an attempt to emphasize each actin monomer, one strand is colored in alternating orange and yellow, while the other in green and blue. B. In order to simplify the view in A, each actin monomer is represented by an orange sphere. The helicity of an actin microfilament can be described by two distinct but equally valid ways: (C) a long-pitched double helix, where the monomers are connected through two silver tubes or (E) a short-pitched single helix, where the actin monomers are connected by a single purple tube rotating in the opposite direction. D. Visual proof for the equivalence of these two different helical descriptions of actin. The “13/6” symmetry of F-actin is derived from the short-pitch single helix description, in which 13 actin monomers are arranged about 6 helical turns (Monomer X is rotationally equivalent to monomer X+13n, where n is any integer). The most recent and highest resolution model of F-actin [16] suggests a slight departure (0.25°/monomer) from the 13/6 symmetry; however, this only amounts to approximately 0.18 Å per monomer at actin's largest radius.
(2.74 MB TIF)

Figure S2 Structural comparison of the previously reported villin crosslink [24] to our proposed model of villin as it exists within the microvillar core bundle. A. The reported structure of villin cross-linking two filaments [24] must be slightly modified because the relative position of the actin filaments (separated by 12.6 nm and offset by 1.7 nm) is not representative of that in the microvillar core bundle. B. Modeled structure of villin cross-linking two actin filaments whose orientation is consistent with that of the microvillar core bundle (12.0 nm apart, without an offset). The new model proposes two new interfaces: (1) Headpiece:V4-6 and (2) V6:Actin, both of which have been previously reported in the literature [54] and [53], respectively.
(4.21 MB TIF)

Figure S3 An alternative model of the fimbrin cross-link [52]. A. Ribbon diagram of fimbrin (blue) cross-linking two actin filaments (orange surfaces). B. When viewed down the long axis of the bundle, fimbrin cross-links exist between every adjacent pair of microfilaments. C. A side view, rotated 90° with respect to B, displays the three distinct vertical levels (d, e, and f) of fimbrin cross-links corresponding to the three different directions of fimbrin cross-links (D, E, and F, respectively). The slight irregularity in the vertical orientation of d, e, and f is a consequence of cross-linking actin's 13/6 symmetry within a hexagonal lattice.
(4.06 MB TIF)

Movie S1 Demonstration that the fimbrin and villin cross-links do not compete with one another. When a centrally located actin filament is excised from the core bundle with every associated fimbrin and villin cross-linker and rotated, it is clear that no steric clashes occur between the two cross-linking proteins because they are vertically staggered in each cross-linking direction.
(3.51 MB MOV)
