# Supplementary figures and images for: Molecular Model of the Microvillar Cytoskeleton and Organization of the Brush Border
Source: PLoS One. 2010 Feb 24;5(2):e9406. doi: 10.1371/journal.pone.0009406 (PMC2827561; doi:10.1371/journal.pone.0009406)

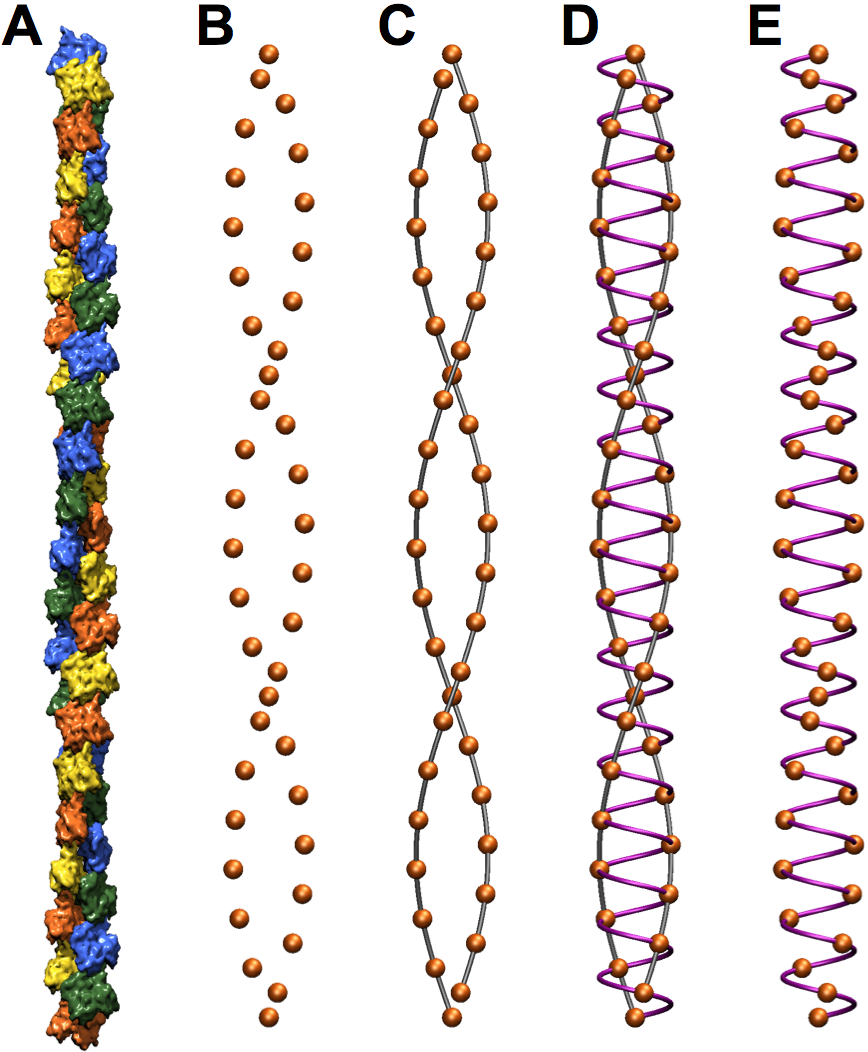

Supplement: Figure S1 — An explanation of F-actin's “13/6” symmetry. A. Surface representation of the F-actin double helix. In an attempt to emphasize each actin monomer, one strand is colored in alternating orange and yellow, while the other in green and blue. B. In order to simplify the view in A, each actin monomer is represented by an orange sphere. The helicity of an actin microfilament can be described by two distinct but equally valid ways: (C) a long-pitched double helix, where the monomers are connected through two silver tubes or (E) a short-pitched single helix, where the actin monomers are connected by a single purple tube rotating in the opposite direction. D. Visual proof for the equivalence of these two different helical descriptions of actin. The “13/6” symmetry of F-actin is derived from the short-pitch single helix description, in which 13 actin monomers are arranged about 6 helical turns (Monomer X is rotationally equivalent to monomer X+13n, where n is any integer). The most recent and highest resolution model of F-actin [16] suggests a slight departure (0.25°/monomer) from the 13/6 symmetry; however, this only amounts to approximately 0.18 Å per monomer at actin's largest radius. (2.74 MB TIF) [file pone.0009406.s001.tif]

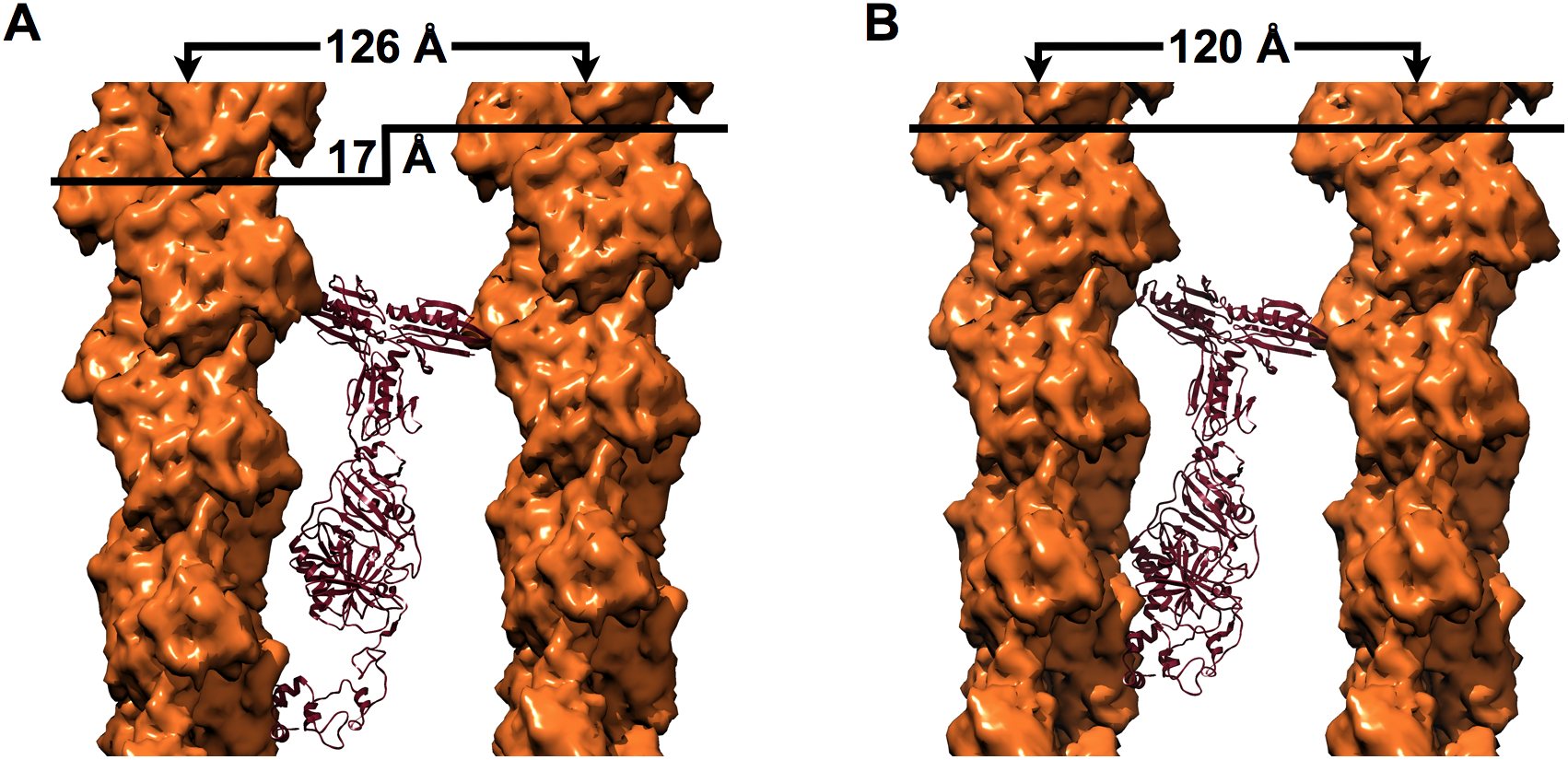

Supplement: Figure S2 — Structural comparison of the previously reported villin crosslink [24] to our proposed model of villin as it exists within the microvillar core bundle. A. The reported structure of villin cross-linking two filaments [24] must be slightly modified because the relative position of the actin filaments (separated by 12.6 nm and offset by 1.7 nm) is not representative of that in the microvillar core bundle. B. Modeled structure of villin cross-linking two actin filaments whose orientation is consistent with that of the microvillar core bundle (12.0 nm apart, without an offset). The new model proposes two new interfaces: (1) Headpiece:V4-6 and (2) V6:Actin, both of which have been previously reported in the literature [54] and [53], respectively. (4.21 MB TIF) [file pone.0009406.s002.tif]

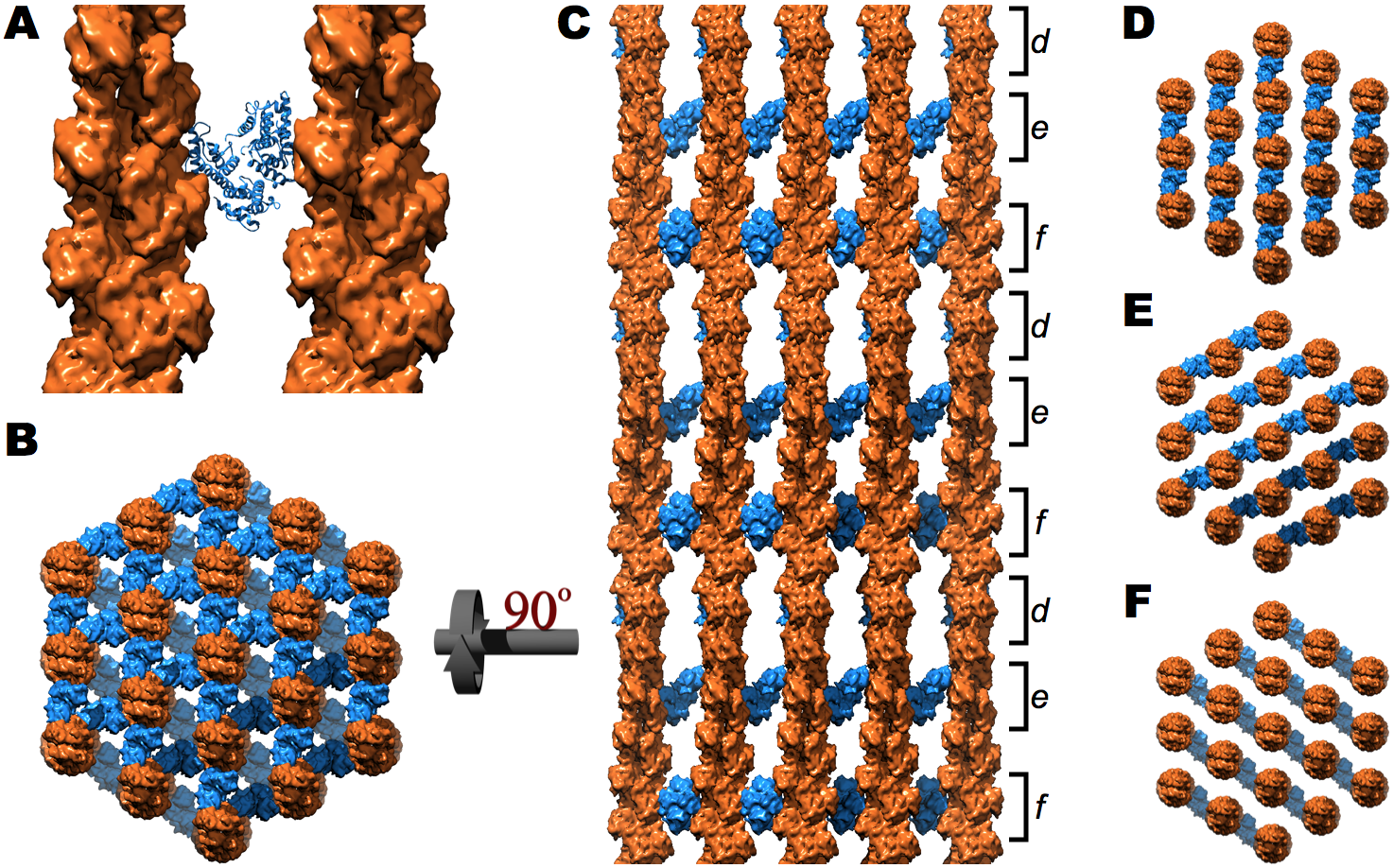

Supplement: Figure S3 — An alternative model of the fimbrin cross-link [52]. A. Ribbon diagram of fimbrin (blue) cross-linking two actin filaments (orange surfaces). B. When viewed down the long axis of the bundle, fimbrin cross-links exist between every adjacent pair of microfilaments. C. A side view, rotated 90° with respect to B, displays the three distinct vertical levels (d, e, and f) of fimbrin cross-links corresponding to the three different directions of fimbrin cross-links (D, E, and F, respectively). The slight irregularity in the vertical orientation of d, e, and f is a consequence of cross-linking actin's 13/6 symmetry within a hexagonal lattice. (4.06 MB TIF) [file pone.0009406.s003.tif]
